# Sinus of Valsalva Aneurysm: Different Modes of Presentation and Techniques of Repair

**DOI:** 10.3390/jcdd11040100

**Published:** 2024-03-27

**Authors:** Thierry Carrel

**Affiliations:** Department of Cardiac Surgery, University Hospital Basel, CH-4032 Basel, Switzerland; thierry.carrel@gmail.com; Tel.: +41-31-351-2410

**Keywords:** sinus of valsalva aneurysm, rupture, surgical repair, interventional repair, aorto-atrial fistula, aortic root repair, Bentall procedure

## Abstract

A sinus of valsalva aneurysm (SVA) is an asymmetrical dilatation of the aortic root located between the aortic valve anulus and the sino-tubular junction. Congenital weakness of the elastic lamina in the aortic media layer or trauma and infection as acquired events are the most principal causes of SVA. Presentation may be acute when rupture has occurred or SVA may be discovered fortuitously on echocardiography or CT scan when patients are examined because of unspecific chest pains, dyspnea or arrhythmias. Although endovascular treatment has been performed successfully in individual cases, surgical closure of the aneurysm aiming at preservation of the aortic valve whenever possible is the established procedure. This short report emphasizes the fact that individual treatment is required when SVA need to be operated, depending on the presentation, the location and the size of the finding. Surgery may consist of simple patch closure, bilateral tunnel closure (entry and exit) or more radical operation like Bentall in case the whole aortic root should be addressed. Overall results are excellent, independently of the clinical presentation (acute or elective) with a mortality approaching zero.

## 1. Introduction

The sinus of valsalva is the anatomic portion of the left ventricular outflow tract located between the aortic valve anulus and the sino-tubular junction. Usually, the wall of the sinus is thinner than that of the ascending aorta; for this reason, it may be subjected to various anomalies, the most common being the sinus of valsalva aneurysm (SVA). The latter should not be mistaken with the more frequently observed aortic root aneurysm, as classically observed in Marfan syndrome patients.

Usually, the SVAs protrude into the adjacent cardiac chambers (right atrium or ventricle, much less frequently left atrium), and the relationship to these structures influences the extension and the location of a potential later perforation. This type of aneurysm may be congenital or acquired and is characterized by the absence of elastic lamellae in the media layer of the aortic wall. Some patients with SVA may present additional defects such as ventricular septal defect, aortic insufficiency and bicuspid aortic valve. Chest trauma, chronic inflammation like vasculitis and iatrogenic injury during aortic valve surgery have been reported as causes of acquired SVA. In this brief report, we would like to summarize different modes of presentation and their individualized techniques of repair.

## 2. Material and Methods

This report summarizes four patients with SVA out of a personal series of 28 cases who presented with completely different symptoms, had rather different clinical and anatomical presentations and underwent an individualized surgical approach to optimally fit with the pathology encountered.

The first patient was a 39-year-old woman who presented with decompensated biventricular heart failure without previous cardiac history. She was complaining about dyspnea NYHA functional class III that appeared suddenly and epigastric tension; at clinical examination, she had hepatomegaly and ascites. A 4/6 systolo-diastolic murmur was auscultated. The liver enzymes were significantly elevated, and the spontaneous INR was 2.2. The transthoracic echocardiography showed a normal sized left ventricle with preserved systolic function (LV-EF 65%) but a heavily enlarged right ventricle and right atrium with moderate tricuspid valve regurgitation and systolic reflux into the hepatic veins. The aortic valve had a trivial central aortic regurgitation. A shunt was suspected between the aortic root and the right atrium with excessive tissue between both structures. Magnetic resonance imaging confirmed the presence of a ruptured sinus of valsalva aneurysm with a Qp/Qs of 1.6.

**Figure 1:**
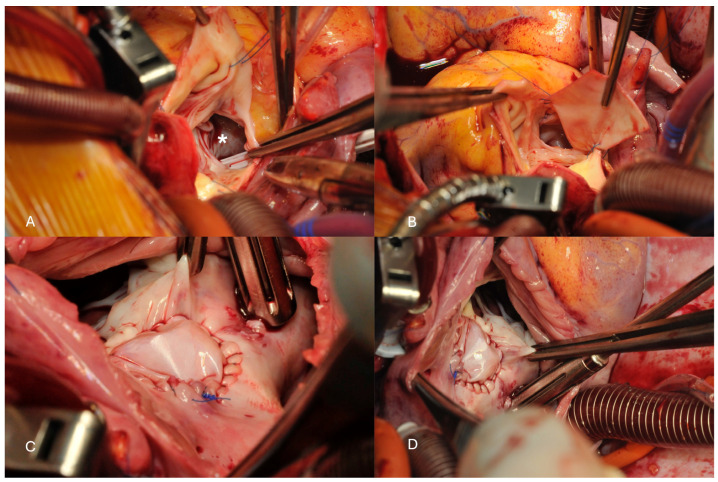
(**A**) View on the aortic root with a large entrance in the sinus of valsalva aneurysm between the aortic sinus and the anterior leaflet of the mitral valve (white *). (**B**) A small piece of xeno-pericardium has been prepared to close the entrance of the non-coronary sinus aneurysm from the aortic root. (**C**) Intraoperative site following closure of the aneurysm on the aortic root side. (**D**) Intraoperative view following closure from the atrial side (through the right atrium, the forceps hold the tricuspid valve).

The repair consisted of a xeno-pericardial patch closure of the defect from inside the aortic root (Figure 1) while both commissures of the aortic valve between the non-coronary cusp and the left, respective right coronary cusp were resuspended with a transaortic stitch reinforced by a small xeno-pericardium pledget. Thereafter, the right atrium was opened and the defect was closed with a second patch on the atrial side.

Weaning from cardiopulmonary bypass was uneventful. The patient was discharged at postoperative day 7. Postoperative echocardiography showed a closed communication between the aortic root and the right atrium and a normal functioning aortic valve. Liver enzymes returned quickly to normal values. Seven years following surgery, the patient is doing well without any episode of heart failure.

The second patient was 50 years of age and suffered from acute chest pain in the early morning. The general practitioner that was consulted suspected an acute type A aortic dissection. Emergency CT scan showed moderate dilation of the aortic root (48 mm). In the echocardiography, a shunt between the right sinus of valsalva and the right atrium was demonstrated with a pressure gradient of 90 mm Hg. Since the patient was in stable hemodynamic condition, coronary angiography with aortography was performed and confirmed the diagnosis without coronary artery disease.

**Figure 2:**
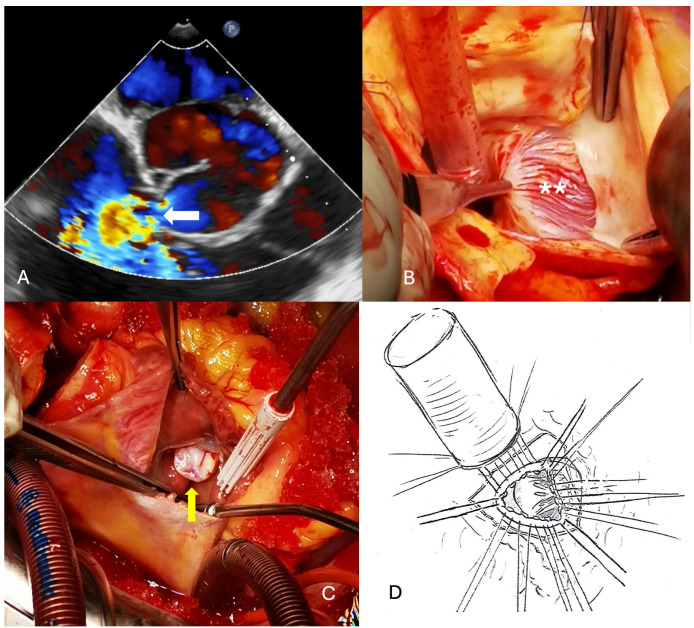
(**A**) Intraoperative echocardiography showing the ruptured sinus of valsalva aneurysm (white arrow). (**B**) Exposure of the aortic wall defect (SVA) (white **) below the right coronary ostium that has been detached. (**C**) Exposure of the defect with the rupture into the right atrium (the aneurysmatic sac is protruding into the right atrium) (yellow arrow). (**D**) Bentall procedure, the patch to reinforce the aortic anulus and the adjacent sinus can be seen under the sutures on the right.

Inspection of the aortic root showed a tunnel-like communication between the right aortic sinus with a distal opening just under the anulus of the tricuspid valve. The aortic and myocardial tissue looked as pulled from each other with a tissue defect of about 1 cm. Inspection within the right atrium showed the distal orifice of the tunnel close to the anteroseptal commissure of the tricuspid valve. The corresponding part of the anulus was missing so that the patch was fixed to the rims of the defect but partially also directly to the tricuspid leaflet (Figure 2). On the aortic side, continuity between the aortic anulus and the aortic tissue was also reconstructed with a xeno-pericardial patch. Because of the diameter of 48 mm, composite-graft replacement was performed with a 25 mm Medtronic tube-valve graft in the classical open technique. Because of the weakness of the tissue close to the aortic anulus, valve-sparing repair after David was not thought to be a good option in this particular case. Weaning from cardio-pulmonary bypass was uneventful as the further recovery. The patient was discharged on postoperative day 6.

The third case was a patient aged 76 years that suffered from a subacute ischemic stroke in the posterior cerebral area and developed quadrant anopsia and optical hallucinations as well as dizziness. He underwent full cardiac investigation to exclude any potential source of embolism. Episodes of paroxysmal atrial fibrillation could not be excluded, but at echocardiography, a large SVA was discovered with suspicion of thrombotic material inside the aneurysm. Cardiac computed tomography confirmed the finding and coronary angiography did not reveal coronary artery disease.

**Figure 3:**
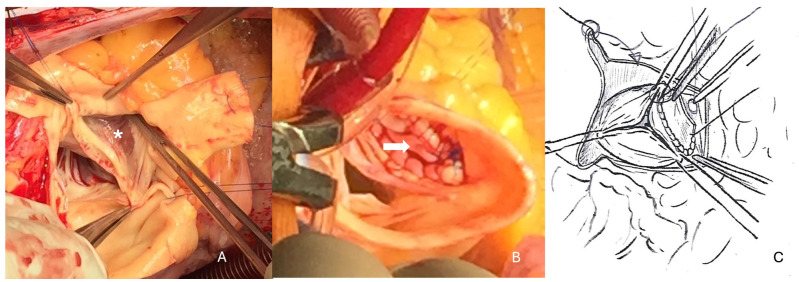
(**A**) Large aneurysm in the right sinus of valsalva (white *). (**B**) The aorta has been transected for better visualization, and the defect closed with a patch of xeno-pericardium (white arrow). This patient received supracoronary aortic graft replacement as well. (**C**) Cartoon with the patch closure below the right coronary artery.

The aorta was transected to better expose the aneurysm that was located below the right coronary ostium. Closure of the cavity was performed using a xeno-pericardium patch with special attention to preserve the geometry of the aortic valve and the commissures. The proximal (ventricular-sided) suture plane was chosen at the level of the anulus, while the distal (ascending aorta-sided) suture line was performed at a level where the aortic tissue was again pretty normal under the right coronary ostium (Figure 3). Because the ascending aorta was of fragile quality and moderately dilated (45 mm), it was replaced with a short vascular graft of 30 mm diameter. Before weaning from cardio-pulmonary bypass, the left atrial appendage was closed with a clip of 40 mm in size.

Postoperative evolution was very favorable; the patient did not demonstrate any neurological deficit and could be discharged at postoperative day 8.

**Figure 4:**
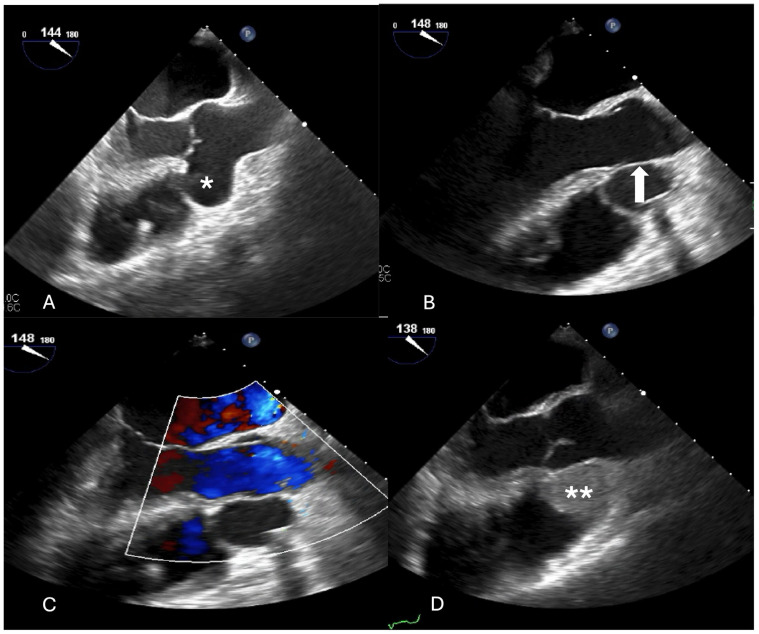
(**A**) Large aneurysm of the right sinus of valsalva with compression of the left ventricular outflow tract (white *). (**B**) Closure with a large patch (white arrow) to exclude the aneurysm (resection not possible). (**C**) Intraoperative transesophageal echocardiography showing normal flow condition in the left ventricular outflow and aortic root without turbulences into the former aneurysm cavity. (**D**) Before closing the chest, the aneurysm cavity had already thrombosed (white **).

The fourth case was a 41-year-old man who presented with palpitations and unspecific chest pain occurring during exercise but at rest as well. ECG showed sinus tachycardia and echocardiography revealed a 5.3 cm SVA of the right sinus protruding into the right ventricle but also into the septum with a moderate narrowing of the left ventricular outflow tract. A CT scan showed no coronary calcifications. The large defect was localized on the left side of the right coronary ostium and closed with a large xeno-pericardial patch from inside the aortic root. Postoperative outcome was excellent, but on first CT scan, there was still a little bit of contrast entering the aneurysmal sack. Six months later, the aneurysm had completely thrombosed and narrowed to a size of about 2 cm (Figure 4).

## 3. Results

Surgery was performed under moderate hypothermic CPB using aortic and bicaval cannulation in all four patients. Myocardial protection was performed following transverse aortotomy via instillation of low-volume crystalloid cardioplegia directly into the coronary ostia (single shot of 100 mL Cardioplexol, Bichsel, Interlaken, Switzerland).

All patients survived surgery and could be discharged without complications within 10 days (6–10 days following the procedure). The further follow-up was very satisfactory with no patient requiring a further intervention.

## 4. Discussion

SVA is a rare congenital lesion caused by the absence of elastic and muscular tissue in the aortic wall of the sinus of valsalva [1]. In a series of 286 patients who presented with a congenital ruptured SVA and underwent surgical repair between 2007 and 2016, SVAs originated from the right coronary sinus (79.7%), the non-coronary sinus (19.6%) and the left coronary sinus (0.7%) but ruptured into the right ventricle (58.4%) and the right atrium (41.3%) [2]. As a consequence, SVA ruptured into the right ventricle in 42 patients and into the right atrium in 18 patients. Patients with unruptured SVA are usually asymptomatic or present unspecific symptoms (rhythm disturbances, dyspnea or chest pains), while some patients may present with more life-threatening complications such as myocardial infarction, stroke and aortic regurgitation [3,4,5,6,7]. In the present series, the presentation was rather different: one suffered from heart failure due to intracardiac perforation, the second presented with severe aortic regurgitation due to progressive enlargement of the aortic root with displacement of the aortic commissures, the third patient suffered from a stroke most probably due to thrombotic embolization out of the SVA, while the last one presented with arrhythmias and the last one had moderate LVOT compression.

In accordance with the observations above, the pathophysiology reflects either the compression of surrounding structures or the location of a perforation with a left-to-right shunt as the most usual consequence. The severity of the clinical manifestations depends on the size of the perforation.

In case of perforated SVA, indication to surgery is always given, independently from the severity of symptoms. In the large majority of the cases, surgery is also reasonable for asymptomatic SVA to prevent complications associated with rupture [8,9]. The recommendations are less clear for small SVA (1–2 cm) with a stable diameter over time.

Although endovascular treatment has been performed successfully in individual cases, surgical closure of the aneurysm aiming at preservation of the aortic valve whenever possible [10]. Nevertheless, two reviews have recently analyzed the outcome of transcatheter closure of SVA: one review included 330 patients from 10 trials (123 in the percutaneous closure group and 207 in the surgical repair group). No statistically significant differences were observed in in-hospital mortality, but as expected, percutaneous closure did significantly decrease the average length of hospital stay. Both methods showed similar and low postoperative residual shunts and aortic regurgitation [11]. The second review analyzed the transcatheter method in 407 patients: the success rate of the trancatheter closure was 95.6% with an overall mortality of 0.5%, but 12% of the patients developed complications, the most significant of which were sustained residual shunts in seven, substantial new onset or progression of aortic insufficiency in six and recurrence of SVA in six [12].

The primary goals of surgical repair of SVA include to close the aneurysm without distorting the corresponding aortic sinus, the aortic anulus or the commissures of the aortic valve. Depending on the location, the aneurysm may be closed through a pericardial patch, and the cavity will be thrombosed or the aneurysmal sack may be removed (for instance when protruding into the right atrium), and the fistula is then closed on both sides. Concomitant aortic valve repair (or replacement) can be performed without difficulty and is mandatory in patients with moderate or severe preoperative AR (grades III and IV). Those with mild preoperative aortic regurgitation (grade II), whose aortic valve geometry is suboptimal, may benefit from aortic valve repair, but in case of a weakened aortic wall, radical replacement of the aortic root may be the better long-term option [13].

In the absence of complicating factors (tamponade, endocarditis), the surgical results of SVA repair are excellent with mortality rates approaching zero. In a series of 286 patients, the most commonly associated deformities were a ventricular septal defect (46.3%) and aortic valve regurgitation (33.2%). The SVA defect was closed using a patch in 90.9% of the patients. Early complications occurred in 5.7% only. There were four late deaths, and the estimated 10-year survival rate was greater than 90% according to the Kaplan–Meier curve [2]. Surprisingly, SVA may also present with unexpected symptoms like arrhythmias very late in life [14].

## 5. Conclusions

This short report emphasizes the fact that individual treatment is required when SVA needs to be operated, depending on the presentation, the location and the size of the finding and consists of simple patch closure, closure of the aneurysm on both sides once it came to a perforation (entry and exit) or more radical operation like Bentall in case the root should be addressed. Long-term results following surgery are excellent.

## Data Availability

Clinical data supporting reported results are saved on the hospital and clinic specific data systems and may be shared upon reasonable request.
